# Supplementary material for: Alkyladenine DNA glycosylase associates with transcription elongation to coordinate DNA repair with gene expression
Source: Nat Commun. 2019 Nov 29;10:5460. doi: 10.1038/s41467-019-13394-w (PMC6884549; doi:10.1038/s41467-019-13394-w)
Supplement: Supplementary file 1 — Supplementary Information [file 41467_2019_13394_MOESM1_ESM.pdf]

Title: **“Alkyladenine DNA glycosylase associates with transcription elongation to coordinate DNA repair with gene expression”**

Montaldo et al.

SUPPLEMENTARY TABLES AND FIGURES

Supplementary Table 1. List of DNA oligonucleotides used for CRISPR-Cas9 editing.

DNA oligonucleotides	Sequence 5' to 3'
AAG$^{-/-}$ sgRNA	GCTGGCGGTGCCTTTCCGGA
AAG$^{-/-}$ sgRNA top	CACCGGCTGGCGGTGCCTTTCCGGA
AAG$^{-/-}$ sgRNA bottom	AAACTCCGGAAAGGCACCGCCAGCC
ELP1$^{-/-}$ sgRNA	AGCTCAGATTCGGAAGTGGT
ELP1$^{-/-}$ sgRNA top	CACCGAGCTCAGATTCGGAAGTGGT
ELP1$^{-/-}$ sgRNA bottom	AAACACCACTTCCGAATCTGAGCAC
HA-ELP1 sgRNA	GACTGAGTGACTGCAGTTAGG
HA-ELP1 sgRNA top	CACCGACTGAGTGACTGCAGTTAGG
HA-ELP1 sgRNA bottom	AAACCCTAACTGCAGTCACTCAGT
PCR #1 HA insertion Fw:	TTGCTCTGACCTGGAGGTTG
PCR #1 HA insertion Rv:	GTAGTCCGGAACGTCGTAG
PCR #2 BspEI digestion Fw:	TTGCTCTGACCTGGAGGTTG
PCR #2 BspEI digestion Rv:	GCTCTCAAACAGCCCAAGTG
HA-ELP1 repair oligo:	TTGCTTTTAGATGCTGAGCTTTTTATACCACCAAAGATCAA<br>CAGAAGAACCCAG<br>TGGAAGCTGAGCCTGCTAGACTACCCCTACGACGTTCCG<br>GACTACGCCTGAGAGAAGACCATTTCCACTCATTCCTGTT<br>GTCCTACCACCCCTTGCTCTTTGAGGGCTGGCTATTGAGA<br>ACTGGAA

Supplementary Table 2. List of DNA primers used to generate mammalian expression constructs containing 1-80aa AAG and Δ80 AAG.

**DNA primers**		
**Name**	**Forward / Reverse**	**Sequence 5' to 3'**
GFP-1-80aa AAG	Fw	TGAGGATCCACCGGATCTAG
	Rv	CTTTGGGCTTGAGAAATAGATGC
1-80aa AAG-GFP	Fw	GATCCACCGGTCGCCA
	Rv	CTTTGGGCTTGAGAAATAGATGC
GFP-Δ80AAG	Fw	GGCCACCTTACCCGACTGG
	Rv	CATAAGCTTGAGCGATCTGAGTCC

Supplementary Table 3. List of DNA primers used for RT-qPCR analysis of mRNA level.

**cDNA primers**		
**Name**	**Forward / Reverse**	**Sequence 5' to 3'**
GAPDH	Fw	GAGTCAACGGATTTGGTCGT
	Rv	TTGATTTTGGAGGGATCTCG
ALDH1A2	Fw	GCTCTCATGGTATCCTCCGC
	Rv	TCCCGTAAGCCAAACTCACC
CRMP1	Fw	CTCTGCTGTCAGTTCGCCAG
	Rv	GATGCGTCCACCTCTGATGA
CDH23	Fw	CGGAGTGGATCCCAGAGAGA
	Rv	GCAAGTGACCAGGGAGTACC
YTHDC1	Fw	GAGGGCCAAATCTCCTACGC
	Rv	GTCTCATGGTCAGAGCCATATTC
SYT9	Fw	GGGATCCAGGAGAACTGTGC
	Rv	TCCGGGTTTGACAAGTTGAGT
CDH4	Fw	GATGTACGTCACAAGGCCCA
	Rv	AGGGCGGTTGTCATTCATGT
NPTX2	Fw	TCTGGTGACTTAAAGGCGCT
	Rv	CTGCACAATGAGACCTCGG
NOVA2	Fw	ACTGTTCCATCACGGCTTTC
	Rv	ACGATGAACCCCGACAGA
CDH22	Fw	CACAACATCACAGTGCTGGC
	Rv	CATACAGCTGCCTCGTAGGG

Supplementary Table 4. List of DNA primers used for ChIP analysis.

**ChIP primers**		
**Name**	**Forward / Reverse**	**Sequence 5' to 3'**
ALDH1A2 -0.5 kb	Fw	GAAGTCGAGCGAGGGTCG
	Rv	CTCTACTCCGAAGCAGCACC
ALDH1A2 +45 kb	Fw	CTGACAGAATTTATCTGAAGGACTC
	Rv	ATGAGGTAAAACTGACCATAGAAGC
ALDH1A2 +111 kb	Fw	GGATGGGAAGAAAGGGAGGC
	Rv	GCCAAGTTCCATTGTGCCAG
CRMP1 -0.5 kb	Fw	GTGGTAAGGGCCAGGAAAGG
	Rv	TGTGTCTGTCGGAGTGTGC
CRMP1 +1 kb	Fw	AATTCGAAAGCTCCCCAGCC
	Rv	GTAGCCTTTGGATGGACCGA
CRMP1 +32 kb	Fw	AGAAGTCATCAGCCGCAGTC
	Rv	CGGATGGTTATTCCCGGAGG
CRMP1 +72 kb	Fw	CAGGTCAGTGGGCAGTTCAT
	Rv	GTTGACGGGGCAGTCAGATT
CDH23 -0.5 kb	Fw	GTTGAGGCCCAGAAAGTCTCA
	Rv	TTCCGAGCTGTCACTGTTCC
CDH23 +1 kb	Fw	CTTTTGCGGCTCTCGCTTC
	Rv	GCTGACCTACGGTGGAGATG
CDH23 +167 kb	Fw	CTCCTTGCCAGGCCCATTTA
	Rv	TATGTTTCATGCCCTGCGGT
CDH23 +420 kb	Fw	CCCCACGTGGACAAGAAAGT
	Rv	AACCGGAGGGAACATCAGTC
YTHDC1 -1 kb	Fw	AGCAGACCATCGAGGAATCG
	Rv	TGTTTCCGTTGGACAATGAATCT
YTHDC1 +1 kb	Fw	AGAGCCCACTTAACAACCCC
	Rv	AATATGACACGGTCGCTGCT
YTHDC1 +17 kb	Fw	AGCCATTATCACACAAAGGGGT
	Rv	AGCCTTCCAGGTTGAATGGG
YTHDC1 +35 kb	Fw	ATCGGCGGAGAGAACAATCC
	Rv	ACCATGTCAGCATATTACCTTCTGT

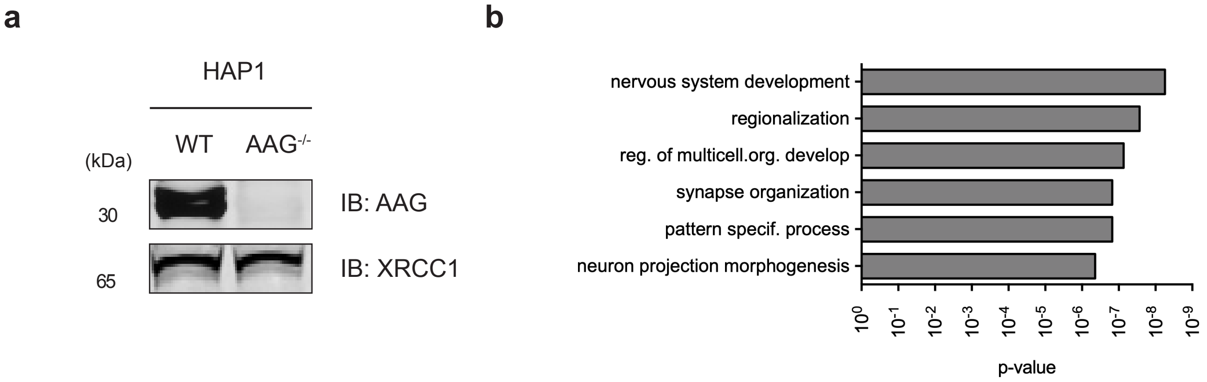

Supplementary Figure 1. *Loss of AAG alters expression of neurodevelopmental genes in HAP1 cells*. (**a**) Immunoblot of whole cell extracts from HAP1 WT and AAG$^{-/-}$ cell lines generated via CRISPR-Cas9 technology. (**b**) Top six biological processes (BP) gene ontology (GO) terms as determined by the Database for Annotation, Visualization and Integrated Discovery (DAVID) for genes dysregulated in AAG$^{-/-}$ in HAP1 cells.

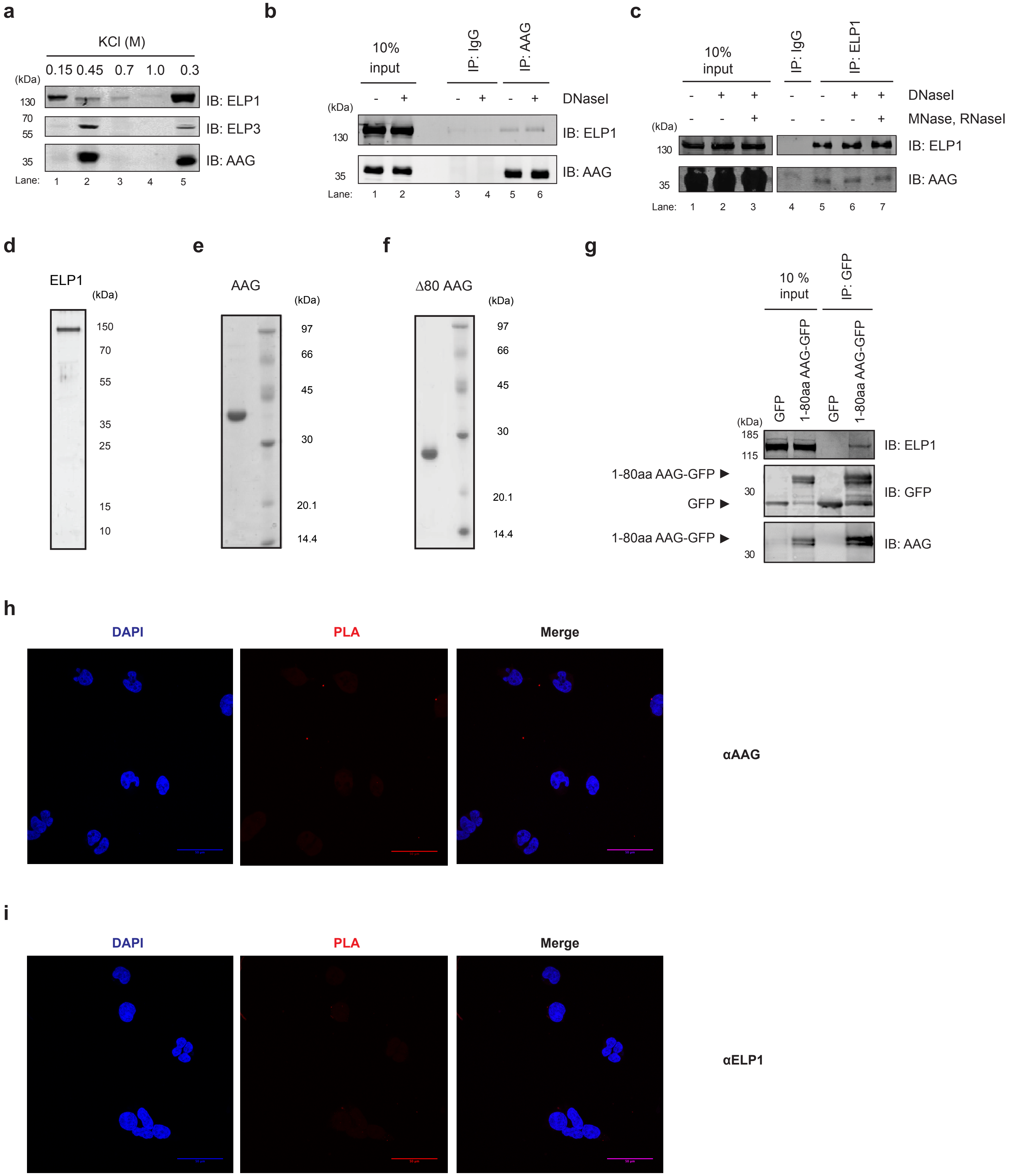

Supplementary Figure 2. *AAG and ELP1 subunit of transcriptional Elongator complex directly interact*. (**a**) Separation of cellular complexes from HeLa cells by heparin-sepharose affinity chromatography. The elutions at the indicated potassium chloride (KCl) concentrations were immunoblotted and probed for AAG and ELP1 and ELP3. (**b**) AAG-mediated immuno-precipitation of whole cell extracts from HEK293T treated and untreated DNaseI. (**c**) IP of AAG from HEK293T WCEs untreated or treated with DNaseI, Mnase and RNaseI. (**d-f**) SDS-PAGE analysis of purified recombinant FLAG-tagged ELP1 (**d**); AAG (**f**); and Δ80 AAG (**e**). (**g**) IP of GFP-tagged first 80 N-terminal amino acids of AAG (1-80aa AAG) (GFP-1-80aa AAG) expressed in HEK293T AAG$^{-/-}$ cells. GFP-tag positioned C-terminally. (**h,i**) Negative controls of prox-imity ligation assay (PLA) in the presence of single antibodies targeting AAG (**h**) and ELP1 (**i**). Scale bar: 50 μm. Source data are provided as a Source Data file.

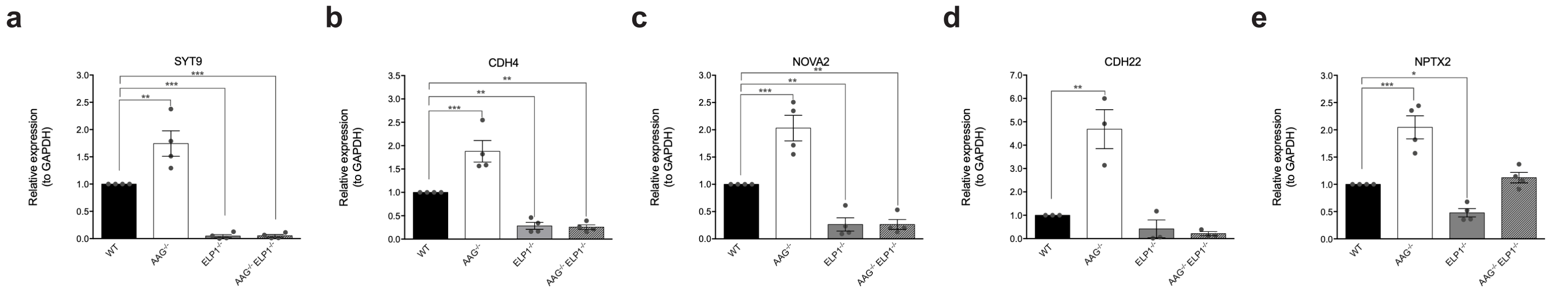

Supplementary Figure 3. *AAG and ELP1 regulate expression of neurodevelopmental genes*. (**a-e**) Expression of additional neurodevelopmental genes *SYT9* (**a**), *CDH4* (**b**), *NOVA2* (**c**), *CDH22* (**d**), *NPTX2* (**e**) in WT, AAG$^{-/-}$, ELP1$^{-/-}$ and AAG$^{-/-}$ ELP1$^{-/-}$. Error bars indicate mean ± SEM (n≥3). *p≤0.05, **p≤0.01, ***p≤0.001, one-way ANOVA. Source data are provided as a Source Data file.

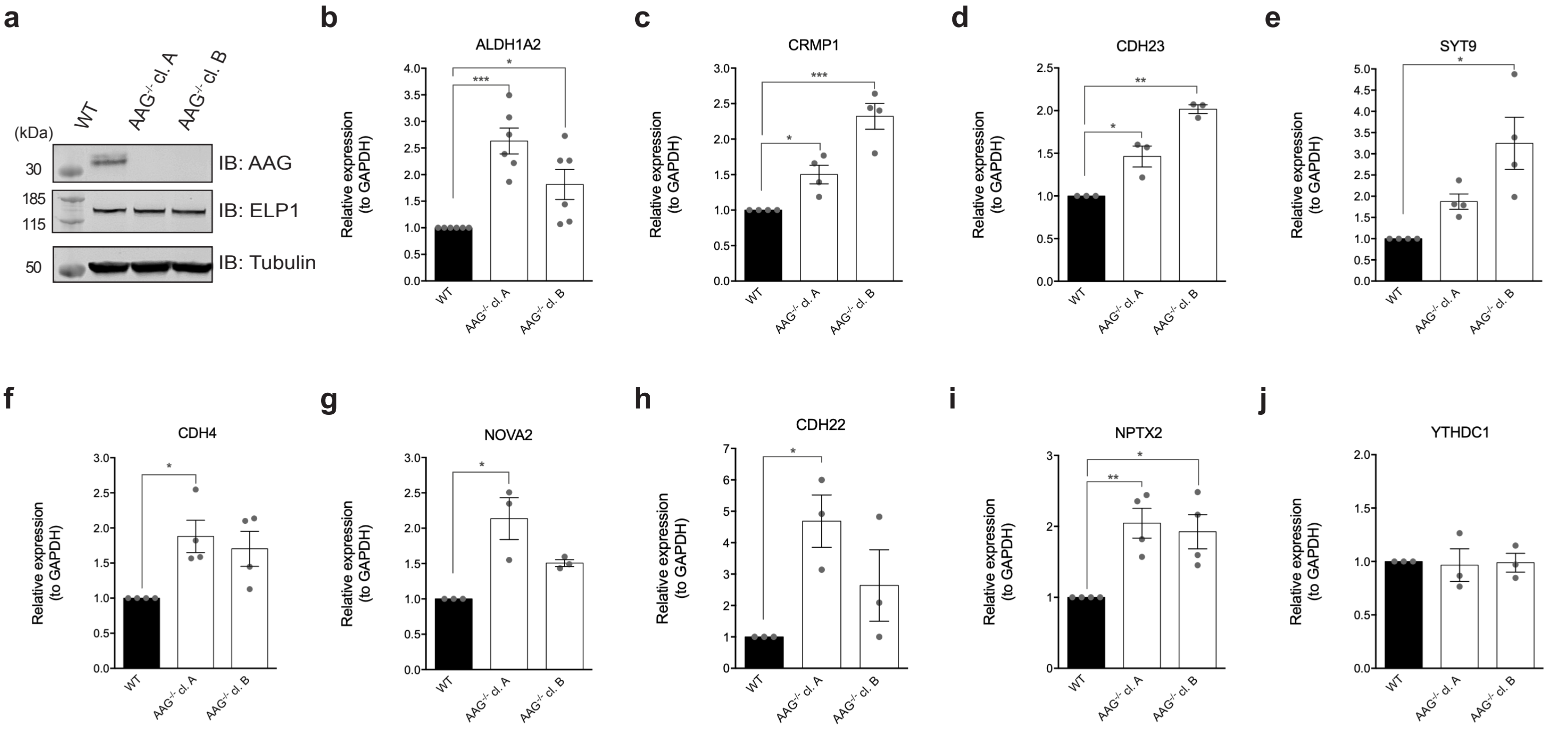

Supplementary Figure 4. *AAG regulatory effect on expression of neurodevelopmental genes is specific and reproducible in different knockout clones*. (**a**) Immunoblot of whole cell extracts of HEK293T WT, AAG$^{-/-}$ clone A and AAG$^{-/-}$ clone B generated via CRISPR-Cas9 technology. (**b-j**) mRNA expression levels of *ALDH1A2* (**b**), *CRMP1* (**c**), *CDH23* (**d**), *SYT9* (**e**), *CDH4* (**f**), *NOVA2* (**g**), *CDH22* (**h**), *NPTX2* (**i**) and *YTHDC1* (**j**) genes in WT, AAG$^{-/-}$ clone A and AAG$^{-/-}$ clone B. Error bars indicate mean ± SEM (n≥3). *p≤0.05, **p≤0.01, ***p≤0.001, one-way ANOVA. Source data are provided as a Source Data file.

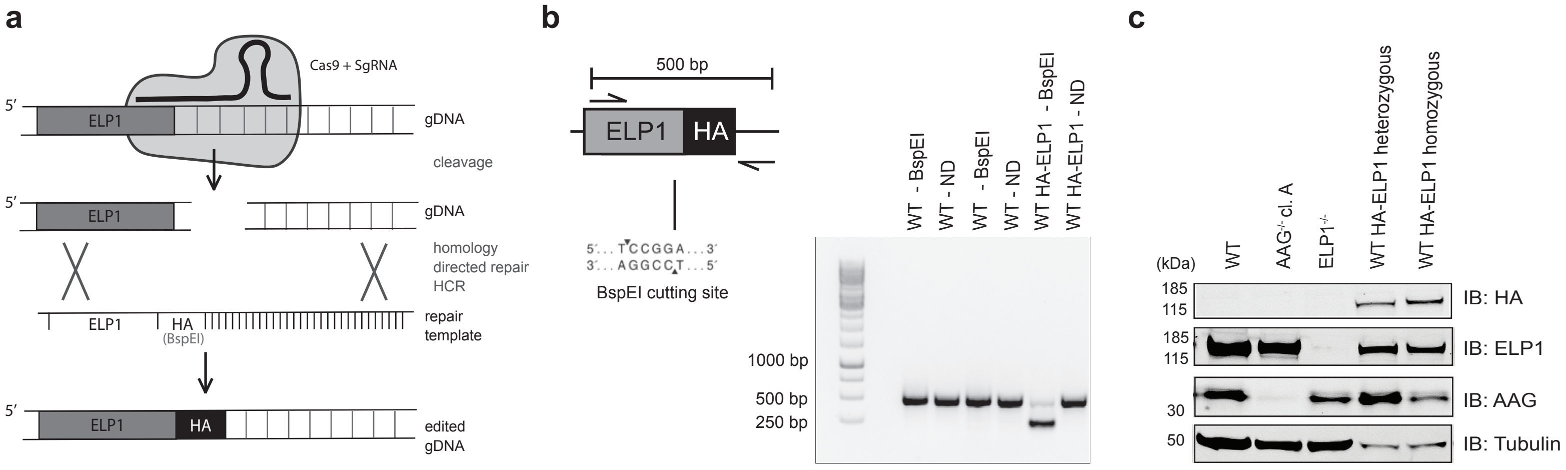

Supplementary Figure 5. *Generation of HA-ELP1 HEK293T cell lines*. (**a**) Schematic representation of the used approach. (**b**) Identification of the clone homozygous for the HA insertion, by PCR and subsequent enzymatic digestion with BspEI. (**c**) Immunoblot of whole cell extracts of HEK293T WT, AAG$^{-/-}$, ELP1$^{-/-}$ and WT HA-ELP1 (heterozygous and homozygous) cell lines generated via CRISPR-Cas9 technology. The HA-ELP1 cell lines tested by immunoblotting were additionally sequenced confirming the HA insertion in frame with ELP1. Source data are provided as a Source Data file.

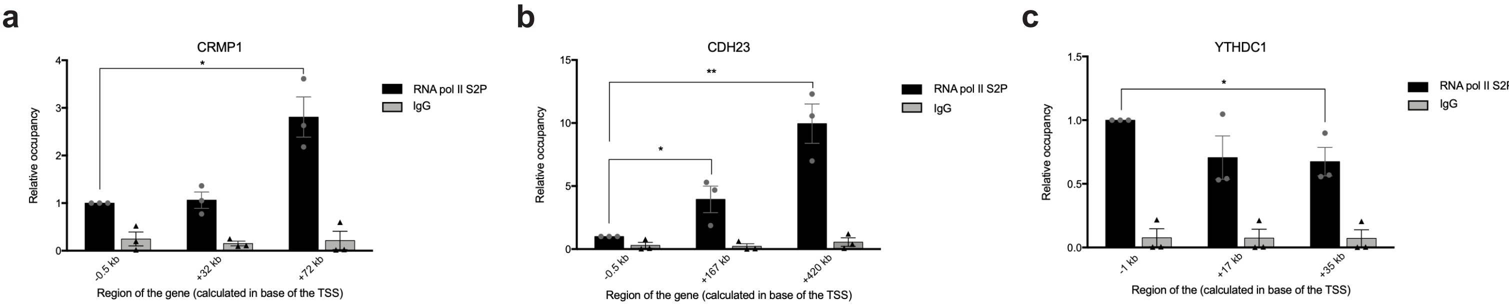

Supplementary Figure 6. *RNA polymerase II distribution along co-regulated genes*. (**a-c**) ChIP assays showing relative RNA pol II S2P (RNA pol II phosphorylated at Serine 2 of the C-terminal domain) occupancy in genes co-regulated by AAG and ELP1: *CRMP1* (**a**), *CDH23* (**b**) and unaffected gene *YTHDC1* (**c**) in WT HEK293T cells. Values are shown as relative occupancy: % input of specific gene region relative to % input of promoter region. Error bars indicate mean ± SEM (n=3). *p≤0.05, **p≤0.01, two-tailed Student's t test. Source data are provided as a Source Data file.

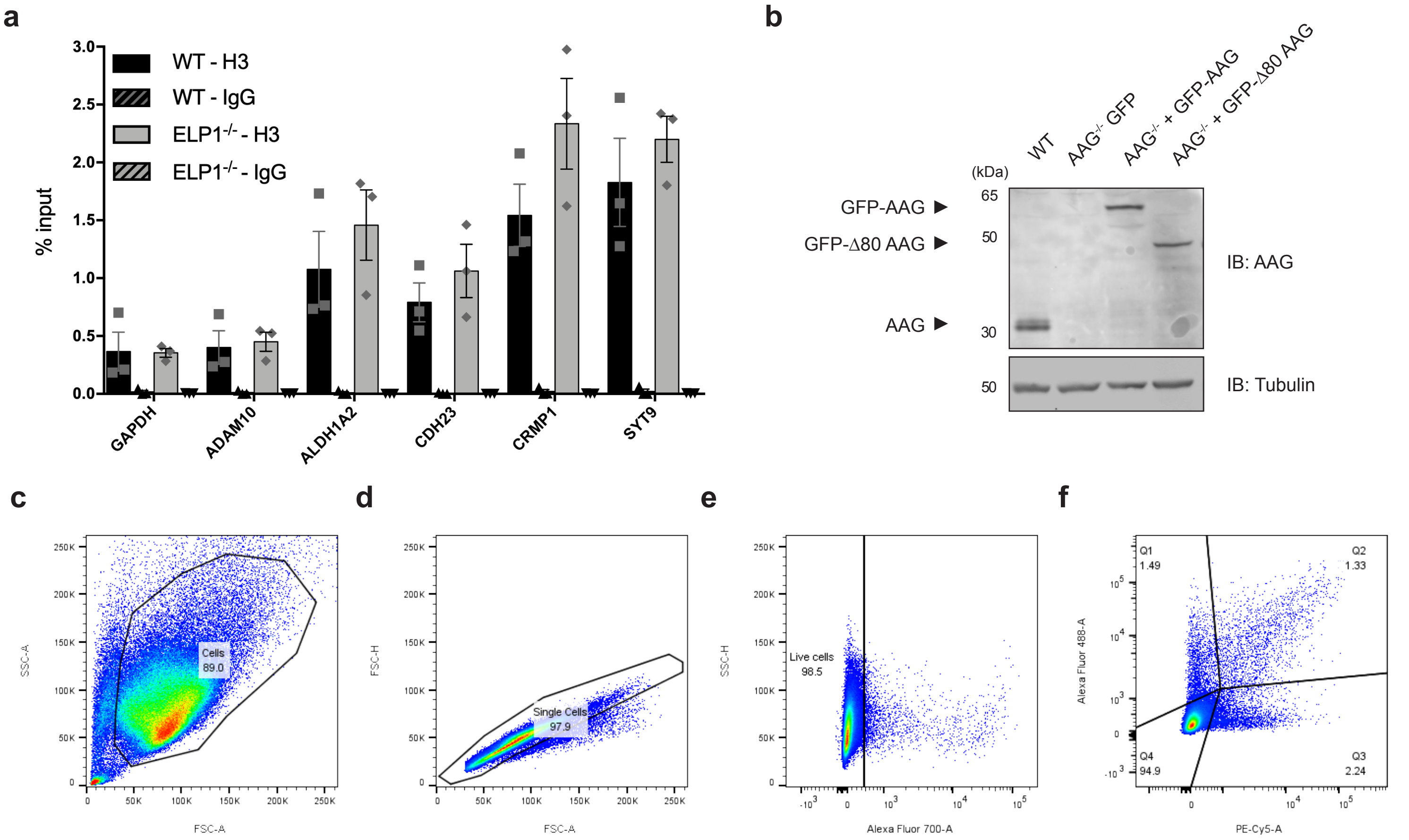

Supplementary Figure 7. *ELP1 status does not affect H3 occupancy, but does influence AAG-initiated BER*. (**a**) ChIP-qPCR experiments targeting histone 3 (H3) in promoters of co-regulated genes in WT and ELP1$^{-/-}$ HEK293T cells. Error bars indicate mean ± SEM (n=3). (**b**) Immunoblot analysis of AAG levels in WT and AAG$^{-/-}$ cells complemented with GFP, GFP-AAG, or GFP-Δ80 AAG. (**c-f**) Representative flow cytometry plots of gating strategies used for FM-HCR presented in Fig.5 i and j to select: cell population (**c**), single cells (**d**), and live cells negative for Zombie NIR (Alexa Fluor 700) (**e**). (**f**) Visualisation of cells positive for GFP (Alexa Fluor 488) and mPlum (PE-Cy5). For the quantification of GFP positive cells Q1 and Q2 were analysed. For the quantitation of mPlum positive cells Q2 and Q3 were analysed. Source data are provided as a Source Data file.

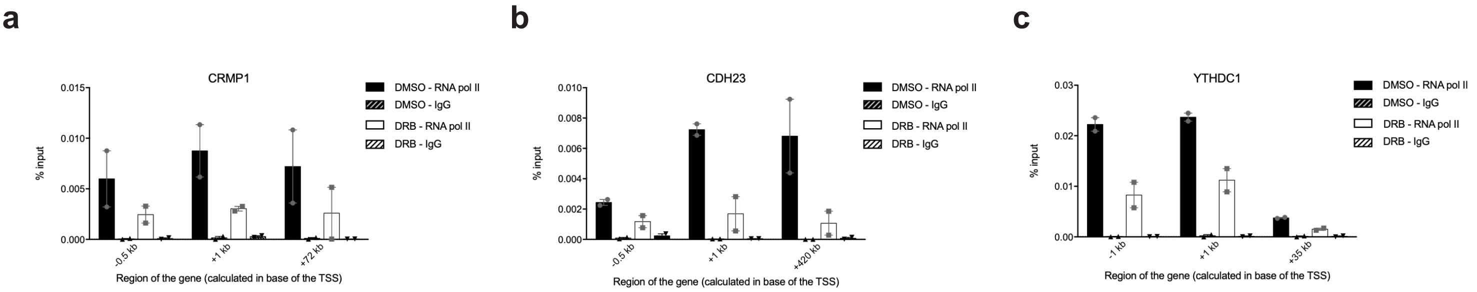

Supplementary Figure 8. *DRB treatment reduces RNA polymerase II occupancy*. (**a-c**) ChIP-qPCR experiments comparing RNA polymerase (pol) II occupancy in DMSO and DRB treated WTCas9 HEK293T cells at *CRMP1* (**a**), *CDH23* (**b**) and *YTHDC1* (**c**) genes. Error bars indicate mean ± SEM (n=2). Source data are provided as a Source Data file.
