# Supplementary material for: Alkyladenine DNA glycosylase associates with transcription elongation to coordinate DNA repair with gene expression
Source: Nat Commun. 2019 Nov 29;10:5460. doi: 10.1038/s41467-019-13394-w (PMC6884549; doi:10.1038/s41467-019-13394-w)
Supplement: Supplementary file 3 — Description of Additional Supplementary Files [file 41467_2019_13394_MOESM3_ESM.pdf]

Description of Additional Supplementary Files

File Name: Supplementary Data 1
Description: Gene ontology (GO) analysis of differentially expressed genes (DEGs) in HEK293T AAG-/-, HEK293T ELP1-/- and HAP1 AAG-/- cells. Depicted are: Count – number of DEGs in each GO category; % – of genes within DEG that segregates to specific biological process; Gene list length for each GO term; and respective p- and Benjamini correction-values.

File Name: Supplementary Data 2
Description: Proteomics Data for FLAG and FLAG-AAG co-immunoprecipitation.
