# Supplementary material for: Alkyladenine DNA glycosylase associates with transcription elongation to coordinate DNA repair with gene expression
Source: Nat Commun. 2019 Nov 29;10:5460. doi: 10.1038/s41467-019-13394-w (PMC6884549; doi:10.1038/s41467-019-13394-w)
Supplement: Supplementary file 6 — Reporting Summary [file 41467_2019_13394_MOESM6_ESM.pdf]

nature research

Corresponding author(s): NCOMMS-19-13109-B

Last updated by author(s): Nov 3, 2019

Reporting Summary

Nature Research wishes to improve the reproducibility of the work that we publish. This form provides structure for consistency and transparency in reporting. For further information on Nature Research policies, see Authors & Referees and the Editorial Policy Checklist.

Statistics

For all statistical analyses, confirm that the following items are present in the figure legend, table legend, main text, or Methods section.

n/a	Confirmed	
☐	☒	The exact sample size (*n*) for each experimental group/condition, given as a discrete number and unit of measurement
☐	☒	A statement on whether measurements were taken from distinct samples or whether the same sample was measured repeatedly
☐	☒	The statistical test(s) used AND whether they are one- or two-sided<br>*Only common tests should be described solely by name; describe more complex techniques in the Methods section.*
☒	☐	A description of all covariates tested
☐	☒	A description of any assumptions or corrections, such as tests of normality and adjustment for multiple comparisons
☐	☒	A full description of the statistical parameters including central tendency (e.g. means) or other basic estimates (e.g. regression coefficient) AND variation (e.g. standard deviation) or associated estimates of uncertainty (e.g. confidence intervals)
☐	☒	For null hypothesis testing, the test statistic (e.g. *F*, *t*, *r*) with confidence intervals, effect sizes, degrees of freedom and *P* value noted<br>*Give P values as exact values whenever suitable.*
☒	☐	For Bayesian analysis, information on the choice of priors and Markov chain Monte Carlo settings
☒	☐	For hierarchical and complex designs, identification of the appropriate level for tests and full reporting of outcomes
☒	☐	Estimates of effect sizes (e.g. Cohen's *d*, Pearson's *r*), indicating how they were calculated

Our web collection on statistics for biologists contains articles on many of the points above.

Software and code

Policy information about availability of computer code

Data collection	Immunoblot (Oddysey LiCor Biosciencies); RNAsequencing (STAR v2.4.2a aligner, Bioconductor (3.2) package Rsubread (1.20.6), and DESeq2, Bowtie2, BWA, RSEM); MS-MS (Sequest, SRF v3); FM-HCR (BD FACSDiva v6.1); qPCR (StepOnePlus v2.3); PLA microscopy (ZEN v2.5, Zeiss)
Data analysis	Immunoblot (GelEval 1.35); RNAsequencing (David v6.8); MS-MS (Scaffold v4.3); statistical analysis (GraphPad Prism 5); Comet assay (Comet IV v4.2); FM-HCR (FlowJo v.10.6.1); model (Biorender); PLA (ImageJ)

For manuscripts utilizing custom algorithms or software that are central to the research but not yet described in published literature, software must be made available to editors/reviewers. We strongly encourage code deposition in a community repository (e.g. GitHub). See the Nature Research guidelines for submitting code & software for further information.

Data

Policy information about availability of data

All manuscripts must include a data availability statement. This statement should provide the following information, where applicable:

- Accession codes, unique identifiers, or web links for publicly available datasets
- A list of figures that have associated raw data
- A description of any restrictions on data availability

RNA sequencing experiments are available in GEO database (accession number GSE 129009; GSE129010); MS-MS data are available in PRIDE database (accession number PXD013508); the original blot images and raw data are provided as the Source Data file. Any data that supports the findings of this study is further available from the corresponding author upon reasonable request.

Field-specific reporting

Please select the one below that is the best fit for your research. If you are not sure, read the appropriate sections before making your selection.

☒ Life sciences ☐ Behavioural & social sciences ☐ Ecological, evolutionary & environmental sciences

For a reference copy of the document with all sections, see nature.com/documents/nr-reporting-summary-flat.pdf

Life sciences study design

All studies must disclose on these points even when the disclosure is negative.

Sample size	No sample size calculation was performed. The sample size were chosen based on the previously published work (RNA analysis: Bjorge et.al. Cell Rep., 2015; fractionation, IP and MS-MS: van Loon and Samson, DNA Repair, 2013; ChIP - Gu et.al. Nat Genet., 2015; Lesion assay - Wang et.al. Method Mol Biol., 2016; FM-HCR - Nagel et.al., PNAS, 2014; Comet - Martin-Pardillos, Blood, 2017)
Data exclusions	No data were excluded from data analysis.
Replication	All experiments were repeated at least in two independent experiments. All attempts at replication were successful.
Randomization	For cell transfection, HEK293T cells were randomly assigned to experimental groups. For immunofluorescent imaging after PLA, random cultured HEK293T WT cells were selected. For ChIP experiments, random cultured HEK293T WT cells were collected and chromatin prepared. For Comet assay, random DRB untreated or treated HEK293T WT cells were selected.
Blinding	Not relevant, as there were no such experimental groups in this study.

Reporting for specific materials, systems and methods

We require information from authors about some types of materials, experimental systems and methods used in many studies. Here, indicate whether each material, system or method listed is relevant to your study. If you are not sure if a list item applies to your research, read the appropriate section before selecting a response.

Materials & experimental systems

n/a	Involved in the study	
☐	☒	Antibodies
☐	☒	Eukaryotic cell lines
☒	☐	Palaeontology
☒	☐	Animals and other organisms
☒	☐	Human research participants
☒	☐	Clinical data

Methods

n/a	Involved in the study	
☒	☐	ChIP-seq
☐	☒	Flow cytometry
☒	☐	MRI-based neuroimaging

Antibodies

Antibodies used

anti-APE1 (Abcam, ab194, clone 13B8E5C2)
anti-ELP3 (Abcam, ab96781)
anti-HA (Abcam, ab9110)
anti-Histone H3 (Abcam, ab1791)
anti-IKAP (ELP1) (Abcam, ab56362)
anti-ELP1 (custom rabbit polyclonal antibody, Genosphere)
anti-MPG (AAG) (LSBio, C133325, clone 1E10)
anti-AAG (custom rabbit polyclonal antibody, Covance)
anti-RNA PolII CTD (PAN) (MBL International Corporation, MABI0601)
anti-RNA polymerase II S2P (abcam, ab5095)
anti-Tubulin (Sigma Aldrich, T9026, clone DM1A)
IgG mouse Diagenode C15400001
IgG rabbit Diagenode C15410206

Validation

anti-APE1 (https://www.abcam.com/ape1-antibody-13b8e5c2-chip-grade-ab194.html);
anti-HA (https://www.abcam.com/ha-tag-antibody-chip-grade-ab9110.html);
anti-Histone H3 (https://www.abcam.com/histone-h3-antibody-nuclear-loading-control-and-chip-grade-ab1791.html);
anti-MPG (https://www.lsbio.com/antibodies/mpg-antibody-clone-1e10-elisa-if-immunofluorescence-ihc-wb-western-ls-c133325/136945);
anti-AAG and anti-ELP1 custom antibodies (specificity confirmed using ELISA and control knockout cell lines as negative controls)
anti-RNA polymerase II S2P (https://www.abcam.com/rna-polymerase-ii-ctd-repeat-ysptsps-phospho-s2-antibody-chip-grade-ab5095.html);

anti-Tubulin (https://www.sigmaaldrich.com/catalog/product/sigma/t9026?lang=en&region=US);
IgG mouse (https://www.diagenode.com/en/documents/datasheet-mouseigg-c15400001);
IgG rabbit (https://www.diagenode.com/en/documents/datasheet-rabbitigg-kch-504-250).

Eukaryotic cell lines

Policy information about cell lines

Cell line source(s)	HEK293T (American Tissue Type Culture Collection); HEK293T AAG-/-, HEK293T ELP1-/- and HEK293T AAG-/-ELP1-/- cells were generated in our laboratory; HAP1 WT and MPG-/- cells (Horizon Genomics)
Authentication	Knock-out cell lines were authenticated by sequencing as well as by immunoblotting. Haploid status of HAP1 cells was confirmed by control sorting.
Mycoplasma contamination	Cell lines were regularly tested for mycoplasma via "MYCOPLAMACHECK" provided by Eurofins genomics. All the cell lines were mycoplasma free.
Commonly misidentified lines (See ICLAC register)	N/A

Flow Cytometry

Plots

Confirm that:

☒ The axis labels state the marker and fluorochrome used (e.g. CD4-FITC).

☒ The axis scales are clearly visible. Include numbers along axes only for bottom left plot of group (a 'group' is an analysis of identical markers).

☒ All plots are contour plots with outliers or pseudocolor plots.

☒ A numerical value for number of cells or percentage (with statistics) is provided.

Methodology

Sample preparation	HEK293T cells transfected with reporter plasmids were detached using trypsin. Trypsin was neutralized with FBS containing media and cells were washed twice with PBS prior analysis.
Instrument	BD LSR II cytometer (BD biosciences).
Software	Data was collected using FACSDiva (BD) v6.1 and processed using FlowJo v10.6.1.
Cell population abundance	Post-sort analysis was not performed.
Gating strategy	The gating strategies are outlined in the Supplementary Figure 7 (c-f): cell population (FSC-A, SSC-A); single cells (FSC-A, FSC-H); live cells negative for Zombie NIR (Alexa Fluor 700-A, SSC-H); cells positive for mPlum and GFP (PE-Cy5-A; Alexa Fluor 488-A).

☒ Tick this box to confirm that a figure exemplifying the gating strategy is provided in the Supplementary Information.
